# Nearly half of Ultrio plus NAT non-discriminated reactive blood donors were identified as occult HBV infection in South China

**DOI:** 10.1186/s12879-019-4215-9

**Published:** 2019-07-03

**Authors:** Xianlin Ye, Tong Li, Wen Shao, Jinfeng Zeng, Wenxu Hong, Liang Lu, Weigang Zhu, Chengyao Li, Tingting Li

**Affiliations:** 1 Shenzhen Blood Centre, Shenzhen, China; 2 Department of Transfusion Medicine, School of Laboratory Medicine and Biotechnology, Southern Medical University, Guangzhou, China

**Keywords:** Blood safety, Non-discriminated reactive (NDR), Re-entry policy, Occult hepatitis B infection (OBI), Molecular characterization

## Abstract

**Background:**

Blood donor plasma samples were detected by the Ultrio Plus NAT system for HBV, HCV and HIV-1 in Shenzhen blood center, China. Reactive samples underwent further discriminatory testing of a single virus by the same methodology. A large number of cases of non-discriminated reactive (NDR) donors were found, leaving potential risk of transmitting HBV if not deferrals. This study identified those non-discriminated samples.

**Methods:**

The NDR plasma samples from blood donation screening were detected and classified by additional molecular and serological tests. Molecular characterizations of DNA+ NDR were determined by sequencing analysis.

**Results:**

A number of 259 (0.21%) NDR plasma samples from screening of 123,280 eligible blood donors were detected, which presented a higher rate (91.1%) of anti-HBc reactivity and nearly half (46.7%) of HBV DNA+ that classified as occult HBV infection (OBI). Most OBI strains were wild-type HBV, but some substitutions V168A, S174 N, V177A, Q129R/L/H, G145A/R in S region of genotype B (OBI_B_) and T47K/V/A, P49H/L, Q101R/H/K, S174 N, L175S, V177A, T118 M/R/K, G145R/A/K/E, R160K/N in S region of genotype C (OBI_C_) strains were identified in high frequency.

**Conclusion:**

Nearly half of NDR blood samples were identified as OBI, in which a number of important mutations were detected. NDR donation might have potential risk for HBV transmission, but need to be further investigated.

## Background

Hepatitis B is a viral infection transmissible by transfusion and remains a global major public health issue [1]. Screening of Hepatitis B virus surface antigen (HBsAg) implemented 40 years ago progressively decreased the risk of transfusion transmission. The sensitivity of HBsAg screening assays was considerably improved over time but still limited to detect the pre-seroconversion window period (WP) or samples with very low viral load after decades of chronicity or clinical recovery [2]. The development of HBV nucleic acid testing (NAT) enabled the testing of donated blood for transfusion and the identification of variable prevalence of HBV DNA carriers in asymptomatic donors negative for HBsAg. However, extremely low viral DNA levels in blood donors with occult HBV infection (OBI) were intermittently appeared or not detectable even by highly sensitive individual donation (ID) NAT [3], which made OBI potentially at risk in transfused patients [4]. In comparison, anti-HBc screening can eliminate nearly all chronic or recovered infections, resulting in a decrease in the risk of post-transfusion HBV infection [5]. In some medium/low endemic countries including Canada, France, Germany, Ireland, the Netherlands, Lebanon, Brazil and USA, anti-HBc was mandatorily implemented in blood donation screening. Nonetheless, in areas where anti-HBc prevalence was > 2–5%, the exclusive of anti-HBc positive donors was impractical and might impact sufficient blood supply [6, 7], especially considering China where anti-HBc screening would eliminate at least 36% donations [8]. HBV DNA screening became the main option after HBsAg in these regions naturally.

HBV has been highly epidemic in China, where epidemiological studies showed about 10% prevalence of HBsAg in general population in 1992. To control hepatitis B, Chinese government has implemented infant vaccination as the highest priority in 1992, and resulted in a significant reduction of carrier rate in children from 10 to < 1% over the two decades [9], which definitely could improve the blood safety. Moreover, to further reduce the risk of blood transmitted viruses, China piloted NAT in blood screening in 2010, and from 2015, China has adopted this technique adequately nationwide. With the combination of sensitive HBsAg screening and vaccination program, blood safety in high HBV endemic area where anti-HBc screening remained unsuitable would be controlled. Shenzhen Blood Center (SZBC) began to implement multiplex Minipool NAT as an option in routine blood screening from 2003, and in 2009, we decided to use ID NAT (Ultrio assay) as a mandatory testing for identifying more low-level viral carriers (HBV-DNA 10 IU/ml). By these NAT assays, the window period (WP) and OBI of HBV infection were identified from blood donations described in previous studies [10, 11]. To ensure maximal blood safety, from Feb 2015, SZBC has adopted more sensitive ID NAT (Ultrio Plus) with enhanced analytical sensitivity for HBV DNA detection (HBV-DNA 3.4 IU/ml, and 6.8 IU/per sample individually for 0.5 mL input volume). This decision was made in consideration that it would detect lower levels of virus and therefore would further reduce the transmission risk of blood from donors in the WP or with OBI.

However, donors who were reactive in the initial multiplex assay but non-reactive in the discriminatory assays (NDR) were problematic, as false reactivity cannot be distinguished from possible occult infection showing low or fluctuating levels of HBV DNA in blood or liver without detectable HBsAg. Furthermore, a dilemma raised regarding how to manage the donor when the discriminatory results were negative, even though Ultrio Plus multiplex reactive donation samples were discarded on the basis of the initial result. In order to clarify and evaluate the true infection status of the donations negative for the discriminatory assays, high volume extraction, nested PCR for S and the basic core promoter/pre-core (BCP/PC) and qPCR [12] were performed, HBV infection will be characterized from these non-discriminators in Shenzhen blood donors, ulteriorly an appropriate re-entry policy for such donors will be discussed.

## Methods

### Subjects and samples

All donors in this study have passed the pre-donation questionnaire. Then underwent rapid pre-donation testing at the collection sites for HBsAg (colloidal gold strip method, Abon Diagnostics, Hangzhou, China), ALT (Roche Refletron, Roche Diagnostics Gmbh, Mannhein, Germany) and hemoglobin (Copper sulfate gravity method). Qualified donors (negative for the pre-donation tests) proceeded to donate blood. A total of 123,280 eligible blood donor samples were collected between Jul 2016 and Dec 2017. At the blood center, donations were tested in paralleled with HBsAg (Diasorin S.P.A.-UK Branch and Wantai Diagnostics, Beijing, China), anti-HCV (Ortho Clinical Diagnostics, UK and Lizhu Diagnostics, Zhuhai, China), anti-HIV1/2 (Biorad, Marnes-la-Coquette, France and Wantai Diagnostics, Beijing, China), Syphilis (Diasorin S.P.A.-UK Branch and Lizhu Diagnostics, Zhuhai, China) and alanine aminotransferase (ALT) with a kinetic method (AusBio Biotech., China). All donors were tested individually by NAT for HBV, HCV and HIV-1 genomes with the multiplex Procleix Ultrio plus assay (limit of detection [LOD]: HBV-DNA 3.4 IU/ml). The initial reactive samples with multiplex NAT were repeated testing with discriminatory Procleix Ultrio plus test to identify the virus responsible for NAT reactivity (HBV, HCV or HIV-1). The discriminatory assays’ methodology was the same as the multiplex assays. Donations showing reactivity in the initial multiplex assay but no reactivity in all three discriminatory assays for HBV, HCV or HIV-1, and with negative results for relevant mandatory serological EIA assays (anti-HIV, anti-HCV, HBsAg) were classified as non-discriminators for further detection.

### Supplemental serological testing

HBsAg (LOD: 0.05 IU/mL), anti-HBs (LOD: 2 IU/L), hepatitis B e antigen (HBeAg), anti-HBe, and hepatitis B core antibodies (anti-HBc) of suspicious or non-discriminators samples were tested and quantified by commercially available electrochemiluminescence immunoassay, ECLI (Roche, USA). Anti-HBc reactive samples were re-tested by a domestic EIA kits (Wantai Diagnostics, Beijing, China).

### HBV DNA confirmation of NDRs

HBV DNA was extracted from 2.5 mL of plasma with all NDRs by HighPure Viral Nucleic Acid Large Volume Kits (Roche Diagnostics GmbH), and were confirmed by a combination of qPCR and nested PCR amplifying the BCP/PC and S regions as previously described [11]. Samples reactive for any tests were confirmed as HBV DNA positive. The LOD of the qPCR assay and Nested PCR were 5 and 10 IU/ml (in combination with 2.5 mL extraction volume of plasma, LOD were 2 and 4 IU/per sample individually). The experiments were performed in a standard PCR laboratory to avoid any contamination. To prevent carryover or cross-contamination during the extraction of DNA from plasma and PCR, each step of the procedure was performed in a separate area with dedicated equipments.

### HBV DNA sequencing, genotyping and analysis

The amplified products obtained from the BCP/PC (295 bp) and S regions (495 bp) were purified by AxyPrep DNA gel extraction kit and sent to Shanghai Invitrogen Co., Ltd. for sequencing. Genotype determination was performed according to S sequences by phylogenetic analysis using the MEGA5.1 program, which fragment was informative for genotyping. Amino acid sequences from 124 genotype B (subtype B1-B7) and 95 genotype C (subtype C1-C7) HBsAg+ blood donors from China, Thailand and Malaysia were selected (provided by Dr. Daniel Candotti) [13] as background sequences. Donations’ amino acid sequences were matched with the background sequences, and each amino acid occurred at every position were calculated statistically.

### Ethical considerations

This study was approved by the SZBC ethics committee. All individuals involved in this study signed an informed consent.

### Statistical analyses

Ninety five percent confidence intervals (95% CI) for the observed yield rate was derived using binomial exact proportion method. The 95% detection limit of real time polymerase chain reaction (PCR) assay and nested PCR assays were determined by probit analysis. Categorical variables were compared using Fisher’s exact test, and for continuous variables, the non-parametric Mann-Whitney test. A *p*-value of < 0.05 was used as the cut-off level for significance.

## Results

### Routine screening results of the blood donor samples

**Fig. 1:**
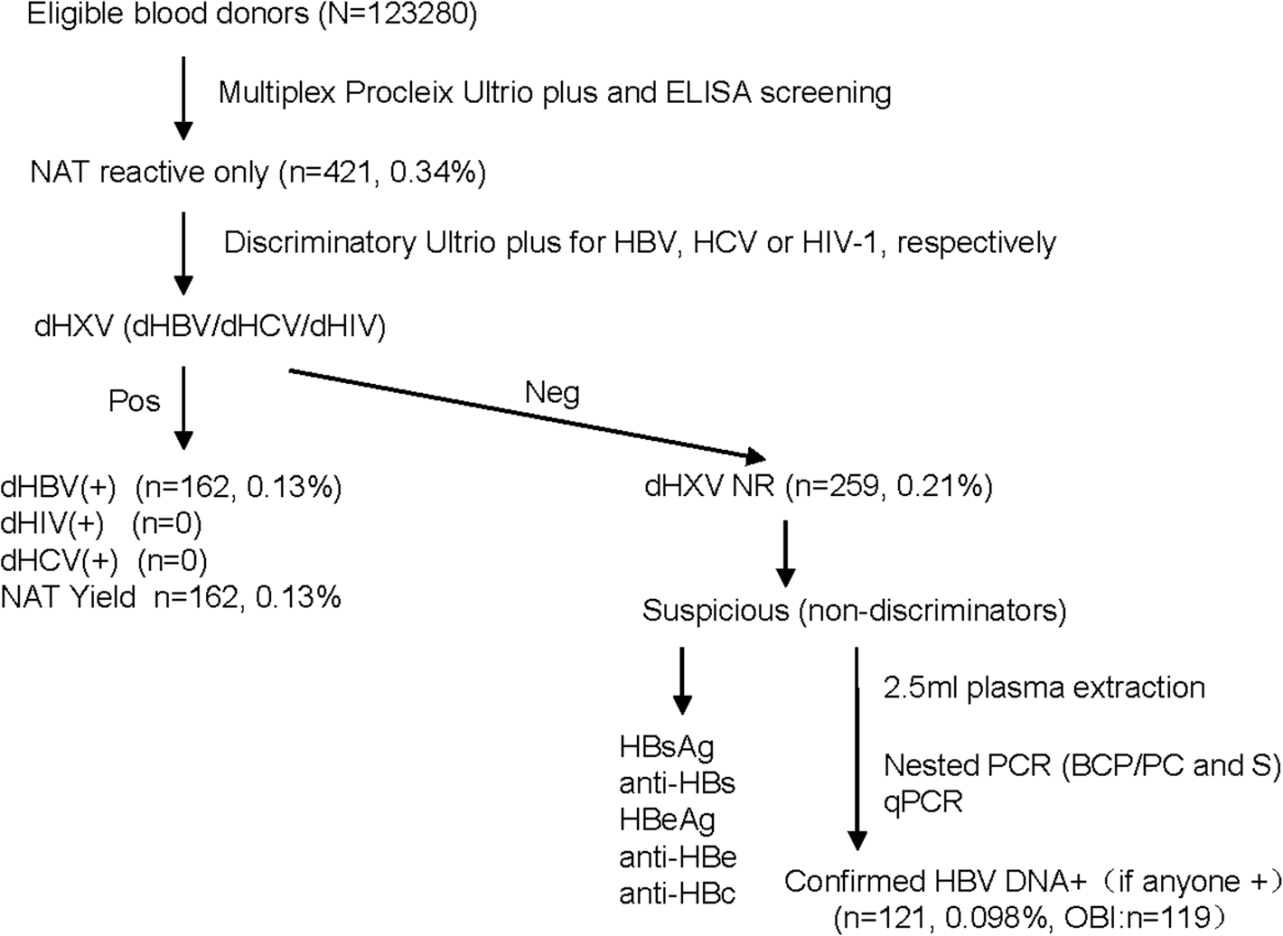
NAT Ultrio Plus screening analysis and confirmatory testing flow

A total of 123,280 eligible blood samples were collected. 513 (0.42%) samples were HBsAg+ plus NAT reactive; 215 (0.17%) samples were HBsAg EIA+ only; and 421 (0.34%) samples were HBsAg, anti-HCV and anti-HIV ELISA negative and Ultrio plus initial reactive, among which 162 donations (0.13%) were reactive for the multiplex NAT and positive for discriminatory HBV test; and the other 259 (0.21, 95% CI: 0.19–0.24%) were multiplex NAT reactive, but negative for discriminatory HBV/HCV/HIV test (non-discriminators). Figure 1 showed the NAT Ultrio Plus screening analysis and confirmatory testing algorithm.

The 123,280 blood donors were distributed in 35.1% females and 64.9% males. And the 259 non-discriminators were 17.4% females and 82.6% males, respectively. The gender distribution between blood donor population and non-discriminators was significantly different (*χ2* = 24.1, *p* < 0.001).

### Supplemental serological testing results in non-discriminators

Despite negative results in the screening ELISA at the blood center, supplemental HBV serologic testing (ECLI) showed that 2 of 259 multiplex NAT non-discriminators (0.77%) were HBsAg positive, with the titers of 0.11 IU/L and 1.73 IU/L, respectively, which were also anti-HBc positive. Table 1 showed that of 259 multiplex NAT non-discriminators, 236 samples were anti-HBc positive (91.1%), 148 samples (57.1%) carried both anti-HBc and anti-HBs, and 88 (34.0%) had only anti-HBc. Seven samples (2.7%) had no detectable serological markers. No HBeAg+ only samples were found. There is no significant difference in anti-HBc and anti-HBs between the NDR DNA pos or neg donations (*p* > 0.05).

**Table 1:** Serological patterns of 259 non-discriminator samples

Anti-HBV	DNA positive			Unclassified
	OBI	WP	Total	
S+/C+	64^a^	/	64	84
S+/C-	6	/	6	10
S−/C+	49^b^	/	49	39
S−/C-	0	2	2	5
Total	119	2	121	138

Number of samples for each group was listed. No HBeAg+ only cases was found. ^a^Included 12 cases with anti-HBe. ^b^Included 14 cases with anti-HBe

### HBV DNA confirmatory testing and classification of 259 non-discriminators

For the 259 non-discriminators screened by Ultrio plus assay, 83 samples (32.0%) were reactive only for one method of BCP/PC, S or qPCR, 33 (12.7%) were reactive for any two methods, and only 5 (1.9%) were reactive for all methods. Totally, 121 (46.7%) NDRs were confirmed HBV DNA+ with any test positive, the rest 138 were unclassified (Table 2). Of 82 (31.7%) qPCR+ donations, the maximum and median viral loads were 65.2 IU/ml and 7.9 IU/ml, respectively. 24 (29.3%) presented VL < LOD.

**Table 2:** Nested-PCRs and qPCR testing results of 259 NDR donations

	Percent					
	BCP/PC+	S+	qPCR+	Any two tests+	Three tests+	Any test+
HBV DNA+	36 (13.9)	46 (17.8)	82 (31.7)	33 (12.7%)	5 (1.9%)	121 (46.7)

### Serological distribution in HBV DNA+ and unclassified samples

Table 1 showed that of 121 confirmed HBV DNA+ samples, 113 samples were anti-HBc positive (93.4%), 64 samples (52.9%) carried both anti-HBc and anti-HBs, 49 (40.5%) had only anti-HBc and 6 (5%) had only anti-HBs. Two were WPs with no markers. Of unclassified 138 samples, 123 were anti-HBc positive (89.1%), 84 (68.4%) carried both anti-HBc and anti-HBs, 39 (28.3%) had only anti-HBc, 10 (7.2%) had only anti-HBs and 5 (3.6%) showed no markers. The sero-markers distributions were not significantly different between HBV DNA+ donors and unclassified donors (*p* = 0.17).

**Fig. 2:**
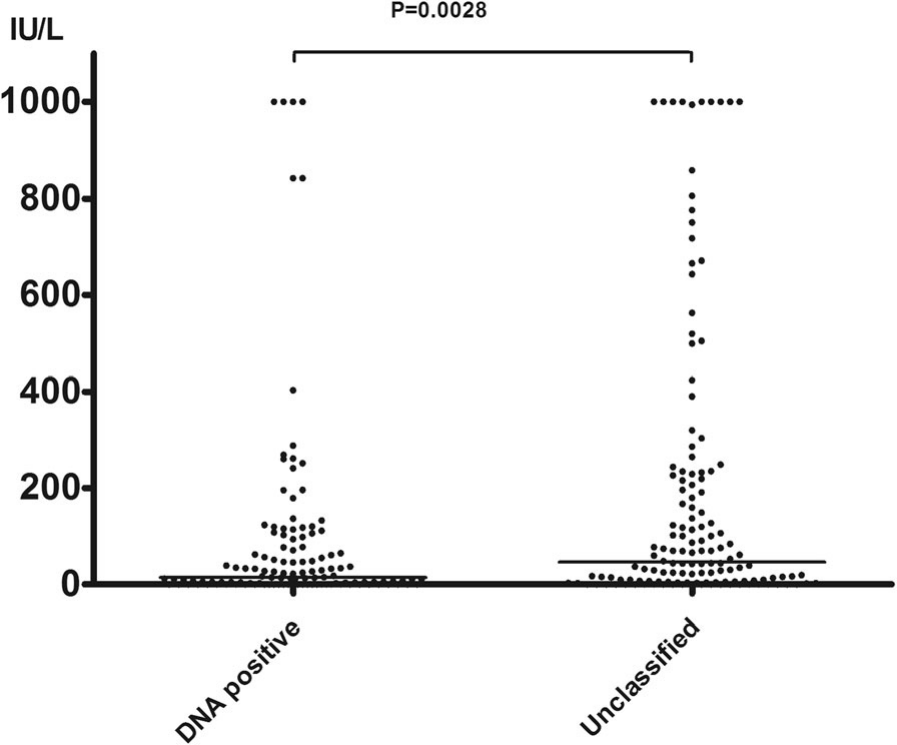
Anti-HBs distribution between DNA positive and unclassified group

The anti-HBs distribution in HBV DNA+ and unclassified samples were further analyzed. Of the 121 HBV DNA+ samples, 51 (42.1%) were negative for anti-HBs, and 70 (57.9%) were anti-HBs positive (> 10 IU/L). Anti-HBs was the only antibody detected in 6 samples (5.0%). 42 (34.7%) had a titer between 10 and 100 IU/L, 21 (17.4%) had a titer within the range of 100–300 IU/L, and 7 (5.8%) had a titer > 300 IU/L (Table 3). For the 138 unclassified samples, 44 (31.9%) were negative, and 94 (68.1%) were anti-HBs positive (> 10 IU/L). Anti-HBs was the only antibody detected in 2 samples (1.4%). 41 (29.7%) had a titer between 10 and 100 IU/L, 27 (19.6%) had a titer between 100 and 300 IU/L, and 26 (18.8%) had a titer > 300 IU/L. The distribution of anti-HBs titer between HBV DNA+ and unclassified samples had a significant difference showed in Fig. 2 (*χ2* = 18.1, *p* = 0.0028). Of 119 confirmed OBIs, 49 (41.2%) were negative for anti-HBs, 70 (58.8%) carried anti-HBs. Only 28 (23.5%) presented a sera anti-HBs > 100 IU/ml.

**Table 3:** Distribution of anti-HBs titers in 259 NDRs according to HBV DNA+, unclassified and OBI

	IU/L				
	Negative (%)	10–100 (%)	100–300 (%)	> 300 (%)	Total (%)
HBV DNA +	51 (42.1)	42 (34.7)	21 (17.4)	7 (5.8)	121 (100)
OBI	49 (41.2)	42 (35.3)	21 (17.6)	7 (5.9)	119 (100)
Unclassified	44 (31.9)	41 (29.7)	27 (19.6)	26 (18.8)	138 (100)

Serum anti-HBs < 10 IU/L were considered negative. *χ*^2^ = 11.15, *P* = 0.011

### Genotyping of NDRs

BCP/PC and S genes were amplified from 259 NDR samples by nested PCRs, 68 donor samples (OBIs) generated 82 HBV sequences, in which 46 S sequences and 36 basic core promoter (BCP)/precore (PC) sequences were included. Phylogenetic analysis of 46 S sequences revealed that 23 (50%) strains clustered with genotype B and 23 (50%) with genotype C.

### Mutation analysis on the BCP/PC in HBV DNA+ donations

The BCP/PC sequences of 36 strains were examined with mutations and related serological patterns presented in Table 4. Nucleotide mutations with high frequency were found. 11/36 (30.6%) strains contained T/G1727A/C mutation in core upstream regulatory sequence, while A1762T/G1764A mutations were observed in 6 (16.7%) sequences. The ATG start codon was abolished by a point mutation (ntA1814C/T) in three cases; and the ntG1896A mutation introducing a stop signal was present in 10 (27.8%) sequences. Combinations of mutations T/G1727A with 1762/1764 were found in OBI092. T/G1727A with G1896A and T/G1727C with G1896A were presented in OBI100/OBI228 and OBI74/OBI185, respectively.

**Table 4:** Classification of 121 HBV+ NDR donations and BCP/PC mutations

Anti-HBV	PCR				Mutations		
	BCP/PC only	S only	BCP/PC + S	total	T/G1727A/C	A1762T/G1764A	G1896A
S+/C+	8	16	7	31	2	4	3
S+/C-	3	1	0	3	1	0	0
S−/C+	10	15	7	33	8	2	7
S−/C-	1	0	0	1	0	0	0
Total	22	32	14	68	11	6	10

Donor OBI092’s combinations of mutations: T/G1727A plus A1762T/G1764A, serological patern of S-C+ with anti-HBe; OBI100 and OBI228’s combinations of mutations: T/G1727A plus G1896A with anti-HBc only; OBI74 and OBI185’s combinations of mutations: T/G1727C plus G1896A with anti-HBc only; OBI079 was A1762T/G1764A with S + C+ plus anti-HBe+; OBI145 was A1762T/G1764A with C+ plus anti-HBe+; OBI043, OBI122 was G1896A with C+ plus anti-HBe +

### MHR or outside of MHR mutations

S sequences including the major hydrophilic region (MHR) of 46 strains (OBIs) were analyzed. The donations’ amino acid substitutions were determined as notable when the occurrence of each substitution was observed with significant difference compared with references (*P* < 0.05) (Table 5). Previously reported HBV vaccine escape variants carrying point nucleotide mutations in the MHR (aa 124–147) of genotype B were also identified including T116 N (4.3%) [15], P120S (4.3%) [16], T126A (8.7%) [17], Q129R/H (17.3%) [18, 19], D144A/E (8.7%) [19] and G145A/R (21.7%) [14]. P120Q/S (8.7%) [21] and Q129H (4.3%) [22] substitutions were detected and recognized to have a major impact on HBsAg detection, while Y100S (8.7%) [23] substitution was observed responsible in OBIs for lack of excretion of HBsAg [24]. A high amino acid diversity was particularly observed in two OBI_B_ donor (7.9 and 7.2%) sequences. Two stop signals at codon s74 and s156 were observed in OBI125 strain. Totally 120 amino-acid substitutions were presented in 23 sequences, with the occurrence frequency significantly higher (*P* < 0.05) than the references of genotype B (256 substitutions/124 strains). In genotype C, vaccine escape related mutations T47K (13.0%) [20], T116 N (4.3%) [20], I126T (8.7%) [20], Q129R (8.7%) [18, 19] and G145A/R (17.4%) [14] were found. P120Q (4.3%) [21], T123A (4.3%) [22], I126S [21], M133 T (8.7%) [21], F134 L (4.3%) [20] substitutions were found which might potentially affect HBsAg detection. Furthermore, two strains showed a 8.5% (14/165) amino acid variability over the whole small S protein. One contained a stop signal codon at s178. Totally 172 amino-acid substitutions were observed in 23 sequences and had a significantly higher occurrence frequency (*P* < 0.05) compared with the references of genotype C (181 substitutions/95 strains). No cysteine or typical P120L substitutions of OBI in the MHR were found in NDRs.

**Table 5:** Notable mutations in MHR and out of MHR of OBIs

Region	Mutation	Frequency (%)
OBI_B_ (Genotype B)		
Out of MHR	S31 N	3/23 (13%)
	Q101R^a^	3/23 (13%)
	M103I	2/23 (8.7%)
	S167 L^a^	3/23 (13%)
	V168A	8/23 (34.8%)
	R169H^a^	2/23 (8.7%)
	S174 N^a^	5/23 (21.7%)
	L175S^a^	3/23 (13%)
	V177A^a^	5/23 (21.7%)
MHR	K122R	4/23 (17.4%)
	Q129R^ab^/L/H^b^	(3 + 1 + 1)/23 (21.7%)
	P142L	2/23 (8.7%)
	D144A^ab^/E^b^	(1 + 1)/23 (8.7%)
	G145A^ab^/R^ab^	(4 + 1)/23 (21.7%)
	S154 L/P	(3 + 1)/23 (17.4%)
	A159V/G	(3 + 1)/23 (17.4%)
	K160 N/R	(2 + 1)/23 (13%)
	E164G/A	(3 + 1)/23 (17.4%)
	Stop codon	2/23 (8.7%)
OBI_C_ (Genotype C)		
Out of MHR	Q30K	4/23 (17.4%)
	S34 L	2/23 (8.7%)
	T47K^b^/V/A	(3 + 3 + 2)/23 (34.8%)
	P49H/L	(5 + 1)/23 (26.1%)
	S55F	3/23 (13%)
	I68T	4/23 (17.4%)
	F79H	3/23 (13%)
	Q101K/R^a^/H/	(3 + 1 + 1)/23 (21.7%)
	V168A	4/23 (17.4%)
	F170S	2/23 (8.7%)
	S174 N	5/23 (21.7%)
	L175S	5/23 (21.7%)
	V177A	5/23 (21.7%)
	Stop codon	1/23 (4.3%)
MHR	P111T/S	(1 + 1)/23 (8.7%)
	T115 N^a^/I	(2 + 1)/23 (13%)
	T116A/P/N^ab^	(2 + 1 + 1)/23 (17.4%)
	S117G/R	(1 + 1)/23 (8.7%)
	T118K/R/M	(4 + 2 + 1)/23 (30.4%)
	K122R	3/23 (13%)
	Q129R^ab^/P^a^	(2 + 1)/23 (13%)
	S136F	3/23 (13%)
	S143 L^a^	3/23 (13%)
	G145R^ab^/A^ab^/K/E	(2 + 2 + 1 + 1)/23 (26.1%)
	I150T	2/23 (8.7%)
	R160K/N	(7 + 3)/23 (43.5%)
	E164D/V/G	(2 + 1 + 1)/23 (17.4%)

^a^OBI related mutations [14]. ^b^vaccination related mutations [14–20]

## Discussion

For the sake of blood safety, many Chinese blood centers adopted the ID-NAT testing strategy of multiplex HBV, HCV, and HIV NAT first, followed by discriminatory assays of HBV DNA, HCV RNA, and HIV RNA after introduction of NAT to blood screening in 2010. Being dependent on the same configuration and the same reagents as the screening assay, the NAT assay cannot be qualified for confirmation [25]. By this strategy, lots of non-discriminators appeared. These donors still keep the right for future donation, raising high risk problem for blood safety. In this study, the occurrence of non-discriminators revealed that 0.21% donors screened using the Ultrio Plus assay in Shenzhen showed NDR results, more than two fold higher than New Zealand (0.09%) [26] and Korea (0.05%) [27], which may be associated with the different prevalence of HBV, distribution of genotype, sampling size of the studies, and the different testing assays and study protocols of different studies. In this study, the percent of anti-HBc reactivity among non-discriminated donations (91.1%) was significantly higher than 47.5% was found in a random group of donors in a previous study (*p* < 0.001), which indicated the background anti-HBc reactivity [28]. It was obviously higher than 13% for Ultrio and 57% for Ultrio Plus in New Zealand [26] and 47% in Korean donors’ population [27]. This strongly indicated that a number of the NDR donors had past HBV infection with low viral load occasionally captured by multiplex HBV, HCV, and HIV NAT, thus missed by discriminatory assays.

The confirmation of low viral load HBV DNA such as NDRs relies on HBV-NAT assays as well as the amount of samples modified to improve sensitivity. Repeat testing with the screening assay from the plasma bag was used in Europe and South Africa in an attempt to discriminate between false and true screening results on the primary test tube and increase the chances of the presence of HBV DNA, but cannot be used for confirmation of HBV infection [25]. Furthermore, anti-HBc was considered in other studies as a critical marker for confirmation [25], but clearly not in this case due to a higher proportion of anti-HBc positive donations involved in NDRs. This study adopted standard nested PCR method targeting different regions of the genome and qPCR with the relatively short amplified region for NDRs’ HBV DNA confirmations. However, these amplification methods may lack the sensitivity required for appropriate confirmation of the screening results with the current triplex NAT blood screening assays. Ultracentrifugation of large volumes of plasma was the first choice in low HBV endemic area [13, 25]. Considering the relatively high background of extremely low level HBV in the anti-HBc + qulified blood donors in China [28], increasing the volume of plasma extracted from 0.5 ml of the screening method input volume to 2.5 ml was proved to be the best choice, highly provided good sensitivity and succeeded in identifying nearly half of the non-discriminators as OBIs by nested PCR or alternative qPCR in this study.

Screening for anti-HBc would have identified and excluded almost all the cases of infectious OBI donors reported. In Brazil, which was also considered high country endemicity, the anti-HBc marker was used for the discard of the bag and permanent refusal of the donor, while the prevalence of anti-HBc was 2.05–6.12% in Brazil blood donors [29]. However, in Shenzhen, China, screening for this marker would exclude more than 40% of blood donors and impair blood supply [28]. Although blood products with NDR were discarded, the donors were eligible to make future donations. Moreover, such number of potential OBI donors would not identified due to the very low viral load and the fluctuation of HBV DNA levels in the future donations [12]. Studies estimated OBI transmission rate for all components varied between 3 and 48% [30, 31], and might be underestimated. In order to detect low level of viral load and avoid transfusion transmission, screening NAT should reach 0.8 copies or 0.15 IU/mL limit of sensitivity [4]. Such high and strict requirement would not be met in a long time. It is urgent to establish an appropriate policy for such donors especially in high epidemic setting. Considering 98.3% HBV DNA+ non-discriminators were confirmed OBIs, even though anti-HBc assay has not be adopted as a universal donor screening test in China, it could be used as re-entry testing for donor with NDR. Assays for HBsAg, anti-HBc and HBV NAT should be performed as part of the re-entry testing on donors deferred due to HBV screening reactivity. Firstly, donors non-reactive for all three assays, or reactive for the anti-HBc assay with anti-HBs > 100 IU/L, and not reactive for the HBsAg assay and HBV NATs, could donate blood in the future. Because it was clearly shown that Ultrio Plus negative donations can be infectious [4], donors with NDR not confirmed but anti-HBc positive might be a safety risk. Only detection of anti-HBs > 100 IU/L appears protective [25]. Necessarily, all donors with NDR should secondly be tested again with an alternative ID-NAT targeting different regions of the viral genome to make sure of no HBV infection to avoid mutation interruption. Deferred donors can be informed to the effect that WP has 95% chance of resolving spontaneously and that this needs to be verified by a physician. Only a donor with confirmation of NDR DNA negative in follow-up detection with an interval of 3–6 months by multiple tests can be informed to be healthy.

The problem with NDR was that the manufacturer of Ultrio Plus stated that the multiplex assay was marginally more sensitive than the discriminatory assay for detecting HBV DNA [27]. This may be why some donations with low levels of HBV DNA produced non-discriminated results. Furthermore, a large proportion of samples had viral load below the 95% LOD of the Ultrio Plus ID-NAT assay [32]. These low viral load units had a high probability of being missed by the subsequent dHBV and this was determined by Poisson distribution [33]. Complementary serological testing of index and follow-up donations might not be sufficient to confirm the infection of non-discriminated donations due to anti-HBc background prevalence especially in high-endemic areas and when there were discrepancies between serological and molecular markers [3]. Additionally, in China where the prevalence of anti-HBc exceeds 40% of the donor population, a positive anti-HBc assay does not provide a reliable adjunct in deciding between a true and false positive HBV DNA result [25]. Such non-discriminated samples with very low viral load were difficult for discriminating from a true-reactive result to a false-reactive one, thus necessitating confirmation of the result with an alternative NAT, by testing a larger volume of samples or nested PCR. A national study reported that 68% ID-NAT Ultrio initial reactive samples were Ultrio test non-discriminated, and only 15% of which showed qPCR+ [32]. A recent study from New Zealand [26] reported that 0.09% of donations tested by Ultrio and Ultrio Plus showed non-discriminated results. These donations were more likely to be anti-HBc reactive (57% for Ultrio Plus) compared with random donors, and at least a third of these donations with anti-HBc were shown to be HBV DNA reactive with an alternative NAT (the Roche Amplicor HBV test) [26]. These measurements would underestimate the real status of HBV infection due to the very low HBV DNA levels that may be only intermittently detectable in OBI donors.

Therefore, more alternative non-exclusive investigational NAT approaches should be considered. In this study, one qPCR and two nested PCRs were adopted simultaneously to investigate the real HBV infection status. Of 259 non-discriminators, 13.9 and 17.8% were obtained for BCP/PC and S sequences, respectively, and finally 26.3% were confirmed positive by nested PCR with sequencing. These donations were considered real HBV infection. In addition, 31.7% were positive by qPCR using 2.5 ml volume of extraction, including 11.2% of which had sequences by nested-PCR. Considering the limited small regions targeted in qPCR and high volume extraction, this result attributed the other potential 20.5% to the final 46.7% of HBV infections, which was more than two-times fold than the previous reports detected by a single alternative assay [26, 32]. More rounds of detections with difference assays could increase the possibility of capturing the virus. A southwest Chinese blood center reported that 84.6% (22/26) of the index donations detected as HBsAg negative but multiplex NAT (Ultrio) reactive with negative discriminatory results were HBV-infected donations after a long time follow up study with multiplex NAT and dHBV assay for dual three repetitions [34]. The discrepancies between this study and previous studies might be related with the alternative NATs and detection times, or different targeting genomic regions. In addition, the number of 91.1% anti-HBc positive donations among NDR donors reflected a proportion of 43.7% HBV past infection in this donor group, after taking the 47.4% anti-HBc positive background off in local blood donors [28]. Further considering anti-HBs only OBIs’ incidence, the rate of anti-HBc in NDR donations consisted with our finding of final 46.7% of HBV infections in NDR donors. More importantly, highly sensitive NATs require more confirmatory algorithms using different genomic regions such as S and BCP/PC avoiding mutations interruption. Non-reactivity in only one assay did not necessarily indicate false reactivity.

In the present study, mutations associated with OBI have been identified with a robust comparison with relevant control cases to exclude natural polymorphism and/or differences related to the geographical origin for blood donors. It firstly provided lots of valuable mutations in envelop protein of OBIs with extremely low level virus. All samples had mutations more frequently than those reported by other studies including Taiwan and Hongkong blood donors’ populations [13, 34]. Some mutations such as Q101R, S167 L, V168A, R169H, S174 N, L175S, V177A, Q129R, D144E/A and G145A/R were in concordance with previous studies [13, 14]. Interestingly, half of the notable mutations in Table 5 were not mentioned before, worthy of further investigation. Although previous studies have already confirmed that the mutations in MHR, especially in the ‘a’ determinant influenced the virus antigenicity and were significantly associated with vaccine escape [14, 22], we observed that the vaccine escaped mutations occurred frequently in the ‘a’ determinant (T116 N (4.3%), P120S (4.3%), T126A (8.7%), Q129R/H (17.3%), D144A/E (8.7%) and G145A/R (21.7%) for OBI_B_ and T116 N (4.3%), I126S (8.7%), Q129R (8.7%), D144E (4.3%) and G145A/R (17.4%) for OBI_C_). Moreover, mutations outside MHR including T47K (13%) and L95 W (4.3%) were also observed in our population. Consistently, a Chinese study reported that a mutation L95 W could impair virion secretion and change antigenicity [35]. Therefore, our data raised an additional issue that should be investigated, if these vaccine escape mutations in the extremely low level OBIs had effect on the risk of HBV.

This study included some limitations. For the increased sensitivity with the 2-stage NAT assay carried increasing difficulties in confirmation, even further evaluation by repeat testing or with an alternative NAT assay didn’t resolve all cases, thus our data would be underestimated. Although the two WPs had been detected repeatedly and got concordant results excluding contamination, we failed in following up the two donors. Also the follow up results to confirm NDR DNA or OBI should have been useful. More sensitive commercial NAT assays such as MPX2.0 ID NAT could interdict more HBV DNA positive NDRs if used in this study.

## Conclusions

In this study, multiple molecular and serological supplemental testing were adopted in NDR donations’ determination and classification, nearly half were identified as HBV DNA+. The results strongly illuminated that a proportion of NDR donors posed an HBV infection risk and a threat to blood safety. At least two NAT assays targeting different regions of HBV genome should be applied in the re-entry policy for such donors. Some mutations including vaccine escape were observed firstly in HBV S gene, further exploration regarding to the function of these mutations should be investigated. This study indicated the urgent need to improve the HBV DNA NAT assays and screening strategies in highly endemic areas so as to decrease transfusion transmitted HBV infection based only on our findings.

## Data Availability

The data used in this study is available from the corresponding author on reasonable request.

## Abbreviations

ALT: Alanine aminotransferase
Anti-HBc: Antibody to hepatitis B Core antigen
Anti-HBs: Antibody to hepatitis B Surface antigen
BCP/PC: Basic Core Promoter/Pre-core
CI: Confidence interval
dHBV: Discriminatory Test for HBV DNA
DNA: Deoxyribonucleic acid
ECLI: Electrochemiluminescence Immunoassay
ELISA: Enzyme linked immunosorbent assay
HBsAg: Hepatitis B virus surface antigen
HBV: Hepatitis B virus
HCV: Hepatitis C virus
HIV: Human immunodeficiency virus
ID: Individual Donation
LOD: Limit Of Detection
NAT: Nucleic Acid Test
NDR: Non-discriminated Reactive
OBI: Occult Hepatitis B virus Infection
PCR: Polymerase Chain Reaction
qPCR: Quantitative real-time PCR
RNA: Ribonucleic Acid
SZBC: Shenzhen Blood Center
WP: Window Period
